# Adolescent mental, sexual, and reproductive health in Ghana: a stakeholder analysis of actors’ influence over policy formulation and implementation

**DOI:** 10.1093/heapol/czaf059

**Published:** 2025-09-10

**Authors:** Emelia Afi Agblevor, Priscilla Ama Acquah, Bernice Gyawu, Lauren Jean Wallace, Tolib Mirzoev, Irene Akua Agyepong

**Affiliations:** Faculty of Public Health, Ghana College of Physicians and Surgeons, P. O. Box MB 429, Accra, Ghana; Faculty of Public Health, Ghana College of Physicians and Surgeons, P. O. Box MB 429, Accra, Ghana; Alliance for Reproductive Health Rights, P.O. Box KD 1012, Kanda, Accra, Ghana; Research and Development Division, Dodowa Health Research Centre, Ghana Health Service, P. O. Box DD1, Dodowa, Accra, Ghana; Department of Global Health and Development, London School of Hygiene and Tropical Medicine, London, Keppel Street, WC1E 7HT, United Kingdom; Faculty of Public Health, Ghana College of Physicians and Surgeons, P. O. Box MB 429, Accra, Ghana

**Keywords:** stakeholders, power, mental health, adolescent health, reproductive health, sexual health, stakeholder analysis, low income, policy process, policy implementation

## Abstract

One in five adolescents (aged 10–19 years) live in sub-Saharan Africa. Despite the availability of policies targeted at this age group, policy formulation, implementation, and gains in adolescent health continue to be underwhelming. Stakeholders or actors are architects of policy, bringing their ideological values, interests, power, and positions to policy formulation and implementation and thus influencing the policy process. We analysed multilevel stakeholder interests, positions, power, and their influence on adolescent mental, sexual, and reproductive health policy formulation and implementation in Ghana, West Africa, using a single-case study design with multiple embedded subunits of analysis. The case was defined as actors, their power, interests, positions, and their influence on policy formulation and implementation processes in adolescent mental, sexual, and reproductive health. A conceptual framework of conflict and synergies between stakeholder interests, power, and positions and their influence on policy formulation and implementation was used to guide the analysis. Data were obtained from key informant in-depth interviews with 19 global and national level and 16 subnational level stakeholders. Focus group discussions were also conducted with 4 district health management teams, 9 groups of frontline health workers, and 20 groups of in and out of school adolescents in four districts in the Greater Accra region of Ghana. The multiple stakeholders in adolescent health, including adolescents themselves, had sometimes synergistic and sometimes divergent and conflicting views on policy agendas, formulation, and approaches to implementation. Unresolved conflicts between powerful stakeholders in the public or bureaucratic arena stalled or hampered policy formulation and implementation, whereas consensus and adequate resourcing moved processes forward. It is important to invest effort in understanding actors, their power, positions, and interests in context to inform policy content and framing to increase the chances of consensus and effective policy formulation and implementation processes.

Key messages

- The formulation and implementation of adolescent mental, sexual, and reproductive health policy in Ghana, a lower-middle-income country with a multiparty democracy, are significantly shaped by actor interests, positions, and power within political, sociocultural, and socioeconomic contexts. The absence of alignment among these factors has led to stalled policies and inconsistent implementation.
- When actors’ values, positions, and use of power align, policy implementation is more likely to succeed. However, divergence and conflict, whether overt or latent, can obstruct policy formulation, delay implementation, or lead to unintended modifications in practice. The absence of early conflict resolution mechanisms has resulted in policies that deviate from their original design.
- Identifying all relevant actors, including latent and non-mobilized stakeholders, is essential. Failure to engage them effectively leads to the emergence of hidden conflicts, policy stagnation, or unpredictable shifts in implementation. Structured dialogue and collaborative approaches can mitigate these risks, ensuring that policy processes are more inclusive and sustainable.

## Introduction

Stakeholders or actors are individuals, groups, or institutions/organizations with an interest in the decisions, actions, and outcomes of the policy process. They are both the architects and agents of any policy process. Stakeholders may serve as initiators, formulators, implementers, supporters, or opposers of a policy. They bring their ideological values, interests, power, and positions that shape policy formulation and implementation processes (Varvasovszky and Brugha 2000, Balane *et al.* 2020, Koduah *et al.* 2022). While studies in low- and middle-income countries (LMICs) have examined actor engagement in adolescent mental, sexual, and reproductive health (AMSRH) (Zhou *et al.* 2020, Ahinkorah *et al.* 2022) and stakeholder perceptions of AMSRH in sub-Saharan Africa (Kumar *et al.* 2018, Arije *et al.* 2022, Okeke *et al.* 2022), fewer studies analyse actor interests, positions, and power and their impact on AMSRH policy formulation and implementation. Existing research highlights the need for further investigation. For example, a qualitative evaluation of the Ghana Education Service’s (GES) abstinence-only policy in Lower Manya Krobo district junior high schools revealed conflicting perspectives and mixed messages among educators and students, limiting effective implementation (Ocran *et al.* 2022).

AMSRH is particularly significant in LMICs, where adolescents (10–19 years) make up about 20% of the population, a proportion projected to rise (Melesse *et al.* 2020, PRB 2021). Adolescence, a period marked by major biological growth and social role transitions (Rutter 1989, Sawyer *et al.* 2012), is increasingly marked by earlier sexual debut and persistently high rates of teenage pregnancy (Zaba *et al.* 2004, Bearinger *et al.* 2007). This trend poses health risks for both the mother and the child while also affecting girls’ education and prospects. In addition, the period of social transition is often accompanied by mental health (MH) issues such as anxiety, depression, stress, academic stress, and suicidal behaviour (Bernard *et al.* 2020, Govender *et al.* 2020, Choudhury *et al.* 2023). Recent studies have found the intersections between MH and sexual and reproductive health (SRH) to be profound, and these intersections exacerbate the risks and vulnerabilities of adolescents’ well-being (Schizoph 2011, Duby *et al.* 2021), underscoring the need for comprehensive approaches to develop and integrate SRH and MH interventions in adolescent healthcare delivery (Denno *et al.* 2015, Desrosiers *et al.* 2020, Kumar *et al.* 2023).

Effective AMSRH policy formulation and implementation require conducive policy environments and active stakeholder participation (OECD 2017, Okoth *et al.* 2023). While diverse perspectives on AMSRH can positively shape the vision and priorities of programmes and interventions, it also raises the potential for conflict when actors’ power, interests, and positions diverge (Ahinkorah *et al.* 2022, Agblevor *et al.* 2023, Obonyo *et al.* 2023).

While previous research has established that stakeholder power, interests, and positions influence health policy processes, this study adds a critical context-specific dimension to existing health policy analysis by focusing on adolescent SRH and MH in Ghana.

In this paper, we ask the specific interrelated questions of ‘Who are the actors involved in AMSRH policy formulation and implementation in Ghana?’ ‘What are their power, interests, and positions in relation to contemporary issues in AMSRH?’, and ‘How do these interact and influence policy formulation and implementation processes?’ Ultimately, we aim to inform health policy analysis in LMICs with a particular focus on adolescent health.

## Conceptual framework

This study draws on conceptualizations of stakeholder power, interests, and position as well as arenas of conflict from the policy analysis literature to organize our enquiry. Conflict, which is essentially a difference of opinion, is inevitable in the policy process, and we drew on Grindle and Thomas’s concept of arenas of conflict as an important characteristic of the policy reform process. Stakeholder reactions to policy formulation and implementation, whether supportive, opposed, or neutral, and conflicts can occur in the public or the bureaucratic arena and can vary across time. Conflict in the public arena is highly visible to society, whereas conflict in the bureaucratic arena is more visible to those within the public bureaucracy and may be invisible to the society (Grindle and Thomas 1989).

Power refers to the ability of an actor to influence other actors (Schmeer 1999). The sources of power manifest in four possible forms: firstly, as resource power where stakeholders control significant financial, human, technological, and political resources, enabling them to allocate or withhold resources as they see fit (Schmeer 1999); secondly, as structural power derived from governance structures, roles, or positions within an institution and includes both governmental and nongovernmental institutions (Mukuru *et al.* 2021, Cruden *et al.* 2023); thirdly, as bureaucratic power derived from the ‘knowledge and authority of bureaucracies’ (Koduah *et al.* 2022); and finally, as technical or expertise power based on specialized knowledge, skills, or relevant expertise (Schneider *et al.* 2022).

Actor interests refer to the concerns, preferences, or objectives that stakeholders hold regarding a particular issue, project, or policy (Schmeer 1999). Stakeholders’ interests can vary widely and may be influenced by a range of factors, including their organizational mandates, financial incentives, values, ideologies, and personal or professional goals (Schmeer 1999). These interests may be explicit or implicit, overt or covert, actual or anticipated, and may align with or diverge from the stated goals of a project or a policy. By understanding stakeholder interests, policymakers and other decision-makers can anticipate potential areas of support or opposition and develop strategies to mitigate conflicts of interest (Agu *et al.* 2022).

Actor positions refer to the explicitly declared or expressed or front-facing support and commitments people make to a particular issue. These positions may also be an opposition, explicitly or implicitly expressed. Stakeholder positions can vary widely and may be shaped by multiple factors, including their interests, values, programmatic ideas, beliefs, expertise, organizational affiliations, and external pressures (Schmeer 1999 , Varvasovszky and Brugha 2000). These positions may be explicitly stated through public statements, declarations, or formal endorsements, or they may be implicitly inferred from stakeholders’ actions, behaviours, or past decisions (Schmeer 1999, Brugha and Varvasovszky 2000).

**Figure 1.**
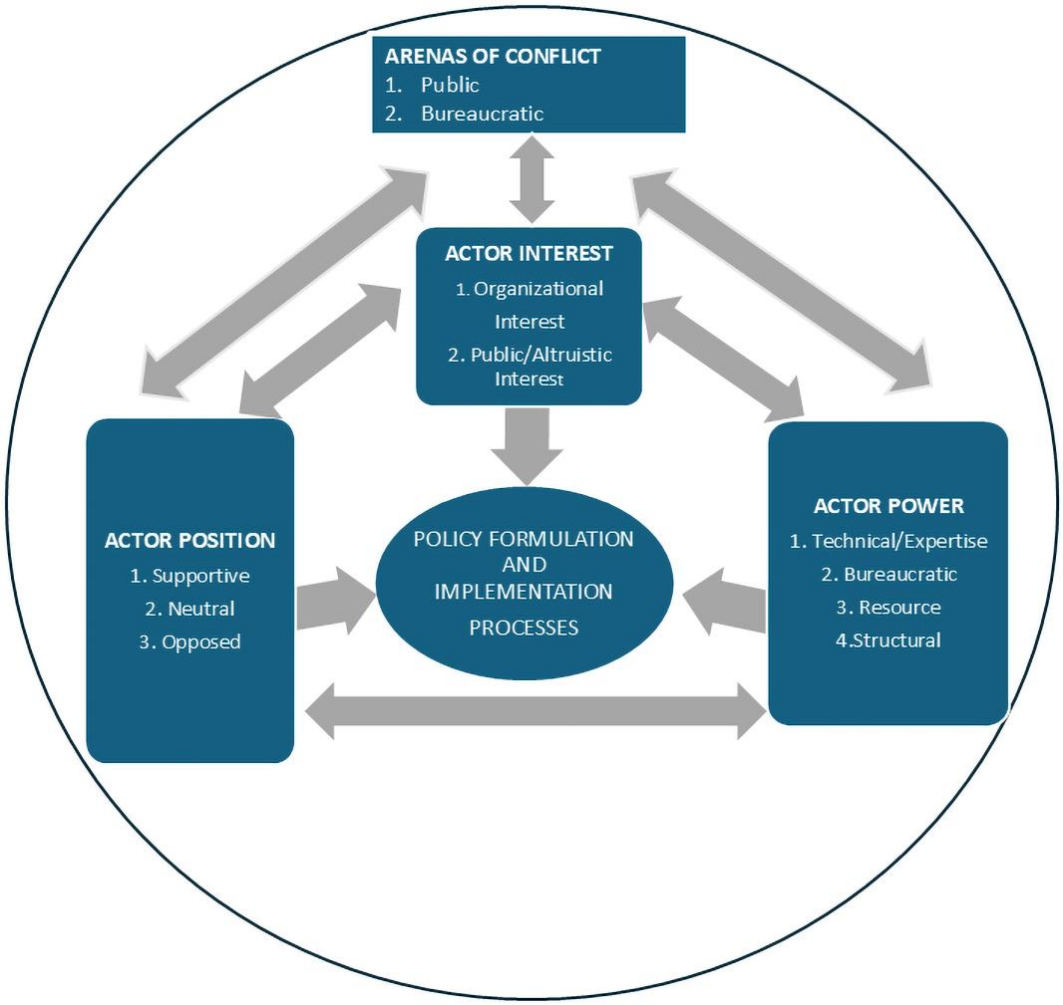
Conceptual framework.

These elements, i.e. interests, position, and power, interact in complex and iterative ways that influence policy formulation and implementation processes and outcomes. Positions informed by interests, shaped by power, and defined by context dictate policy priorities and strategies. Actors exert influence based on their interests, which may not always align with their stated positions. Power dynamics resulting in conflicts both in the public and bureaucratic arena further complicate the process, with power imbalances affecting interactions, decisions, and outcomes (Grindle and Thomas 1989, Béland 2009, Erikson 2015). The conceptual framework is captured in Fig. 1.

## Methods

### Study design

The study design was a single-case study with multiple embedded subunits of analysis. The study context was Ghana, a lower-middle-income country in West Africa. The overarching case was defined as ‘Stakeholder power, interests, and positions and their influence on policy formulation and implementation.’ Each subunit of analysis was a single contemporary public policy issue in AMSRH in Ghana for which policy and programme formulation had been completed, and an attempt had been made to move to implementation, whether the attempt at implementation was successful or not. The selection of the cases was purposive, based on the research questions. Stakeholders were asked to identify AMSRH issues in the 10 years preceding the study (2011–2021). They were then asked to focus on two policies or programmes related to these issues that they had been involved in, either in adolescent SRH (ASRH) or adolescent MH (AMH), where formulation was completed and efforts to implement had started. They were also asked about their specific roles, perceptions as to whether the policies were proving effective in implementation, and priorities that were not being implemented. The seven cases selected were those consistently emerging in discussions with stakeholders.

### Study context

Ghana in West Africa, where this case study was undertaken, is a lower-middle-income country with an estimated gross national income per capita of US$2380 (MacroTrends 2024). While Ghana's economy remains predominantly agricultural, the services sector is growing. Mining, particularly gold, remains significant. The discovery of crude oil offshore has further diversified the economy. Despite steady economic growth and declining poverty rates in recent decades, income inequality has increased (Aryeetey and Baah-Boateng 2016). Politically, the country has maintained a stable multiparty democracy with 4-year election cycles since 1992, fostering an inclusive policy-making environment. However, government financing for health remains inadequate while development assistance for health (DAH) is decreasing due to Ghana’s reclassification as a lower-middle-income country.

Greater Accra, one of the 16 regions, was purposively selected for its diverse sociocultural characteristics, mix of rural and highly urbanized contexts, and accessibility to national and subnational level stakeholders involved in AMSRH formulation and implementation. Its proximity to the research institution also enhanced feasibility within available resources.

### Data collection methods, tools, and analysis

Data were collected between 2021 and 2023 using a desk review of legislative, policy, and administrative documents relevant to adolescent health, key informant interviews (KIIs), and focus group discussions (FGDs). For the desk review, we identified and reviewed 21 documents (see Table 1). The initial documents reviewed were identified from the websites of stakeholder agencies. Additional documents were suggested during the KIIs, and these were subsequently reviewed. The policy and legislative material documents covered the postindependence era (1957) till the time of the study (2023). The documents were analysed inductively using content analysis. The analysis allowed us to view how adolescents were represented in policies, the prioritization of AMSRH issues, and the extent to which policy strategies were multisectoral. We also examined how policy emphasis and commitments evolved over time, with attention to recurring gaps, unaddressed priorities, and implementation challenges.

**Table 1.** Legislative, policy, and administrative documents reviewed.

Description	Year of publication or period covered
**Category: Legislative Instruments**	
The Constitution of Ghana	1992
The Mental Health Decree	1972
Mental Health Act 846	2012
The Children’s Act	1998
**Category: Sexual and Reproductive Health Policy**	
The Ghana Health Service Adolescent Health Programme	1996
Adolescent Sexual and Reproductive Health Policy	2000
National HIV/AIDS and STI Policy	2001
Reproductive Health Strategic Plan	2007–2011
National Reproductive Health Services Policies & Standards	2007–2014
Adolescent and Health Development Strategy Plan	2009–2015
Adolescent Health Service Policy and Strategy	2016–2020
5 Year Strategic Plan to address Adolescent Pregnancy in Ghana	2018–2022
Guidelines for Comprehensive Sexuality Education in Ghana	2019
Integrated Strategic Plan for Reproductive, Maternal, Newborn, Child & Adolescent Health and Nutrition (2020–2025)	2020–2025
**Category: Mental Health Policy**	
Ghana Mental Health Policy	1994
Mental Health Policy	2017–2030
**Category: Cross cutting/Other Policies/Guidelines related to adolescent health in general**	
National Youth Policy	1999
National Population Policy	2001
School Health Education Programme (SHEP) Policy Guidelines	Not Dated
National Operational Guidelines for Adolescent and Youth Friendly Services	Not dated
Policy Guidelines for School-based Health Services	2022

A total of 35 KIIs were conducted with purposively selected respondents. Nineteen KIIs were conducted with global and national level stakeholders and 16 KIIs with subnational level stakeholders. Table 2 displays all the respondents in KIIs by institution, level of operation (global, national, subnational), and numbers interviewed. The initial list of interviewees was drawn from the desk review of legislative, policy, and administrative documents. A snowballing approach was then used to identify additional interviewees by asking initial interview participants to advise on any other stakeholders who needed to be interviewed. All KIIs were conducted in English and held either using Zoom or in person, depending on stakeholder preference.

**Table 2.** Respondents in KIIs by actor category, agency/institution, and level of operation. Comma after institution.

Actor category and agency	Level	Number interviewed
**Category: Government**		
Ghana Health Service	National	3
National Population Council	National	2
Ministry of Gender, Children and Social Protection	National	1
Ghana Education Service	National	1
National Youth Authority	National	2
Mental Health Authority	National	1
Ghana Health Service District health directorate, Adolescent Health focal persons	Subnational	4
Ghana Education Service; District Education directorate, Girl Child Education Officer	Subnational	3
Ghana Education Service; District Education directorate, School Health (SHEP) Coordinator	Subnational	3
Ghana Education Service, District Education directorate, Guidance and Counselling Coordinator	Subnational	2
Social Welfare and Community Development Department Social Welfare Director	Subnational	1
Social Welfare and Community Development Department Social Welfare Officer	Subnational	3
**Category: Non-Governmental**		
Planned Parenthood of Ghana (PPAG)	National	2
Marie Stopes International (MSI)	National	1
Alliance for Reproductive Health Rights	National	1
Basic Needs	National	1
**Category: External Development Partner**		
Foreign Commonwealth Development Office (FCDO)	Global bilateral	1
United Nations Children’s Fund (UNICEF)	Global multilateral	1
United Nations Population Fund (UNFPA)	Global multilateral	1
World Health Organization (WHO)	Global multilateral	1

A total of 23 FGDs were held: four with district health management teams, 9 with frontline health providers, and 20 with adolescents in and out of school in the four study districts. All FGDs were held in person, they were conducted in English,Twi and Ga. All primary data collection were audio recorded and done with informed consent.

The KII and FGD guides were developed by the research team based on the objectives of the study and informed by the desk review as well as an analysis and understanding of the local policy context in Ghana. Different tools were used for different levels of the health system and stakeholders. At the national level, we wanted to understand who the policy actors were and how their ideas, ideology, power, and interests shaped AMSRH policy priorities, formulation, and implementation. At the district, subdistrict, and community levels, we wanted to understand microlevel decision-making processes for implementing AMSRH within adolescent health policies. From adolescents, we wanted to understand their perspectives on the issues as well as their knowledge of service availability, use, and how services could be configured to better respond to their needs. The guides were piloted and refined before final use.

To analyse the KIIs and FGDs, the audio recordings were transcribed verbatim and were quality checked for accuracy. The qualitative data analysis software QSR NVivo (version 20) was used to manage and code the transcripts. Deductive codes were drawn from the study’s conceptual framework on actor interests, power, and positions, while inductive codes emerged from multiple readings of the transcripts. To ensure intercoder reliability, the first and second authors, who also conducted the interviews, coded the transcripts independently. All other authors reviewed the analysis and interpretation, agreeing on the final themes and codes.

Following Marques et al. (2020), we structured our stakeholder analysis into three steps. First, we compiled an exhaustive list of actors from the document review. Second, we collected qualitative data using KIIs and FGDs, which complemented the information on stakeholder power, interests, and positions from the desk review. Finally, we completed a table containing stakeholder power resources, positions, and interests based on all the evidence gathered. Power was conceptualized as having resources and the ability to use them (Schmeer 1999). The stakeholder analysis table, therefore, had various columns corresponding to our conceptualization of power (Schmeer 1999, Varvasovszky and Brugha 2000, Koduah *et al.* 2022, Schneider *et al.* 2022). Under resources, we had financial, human, technological and political resources, structural power, bureaucratic power, and technical/expertise power. These resources were classified as low, moderate, and high (Schmeer 1999). Stakeholder interests were classified as either high or low (Schmeer 1999, Koduah *et al.* 2022). Stakeholder position was classified as supportive, nonaligned, and opposed in reference to the person being interviewed as well as their organization (Schmeer 1999, Marques *et al*. 2020, Koduah *et al.* 2022), allowing us to capture divergences between personal and institutional commitments. The stakeholder analysis table was updated iteratively during the interview process.

The triangulation of data from policy documents, KII interviews with national and subnational level stakeholders, and FGDs with subnational actors and adolescents strengthened the robustness of our analysis and conclusions.

## Results

**Figure 2.**
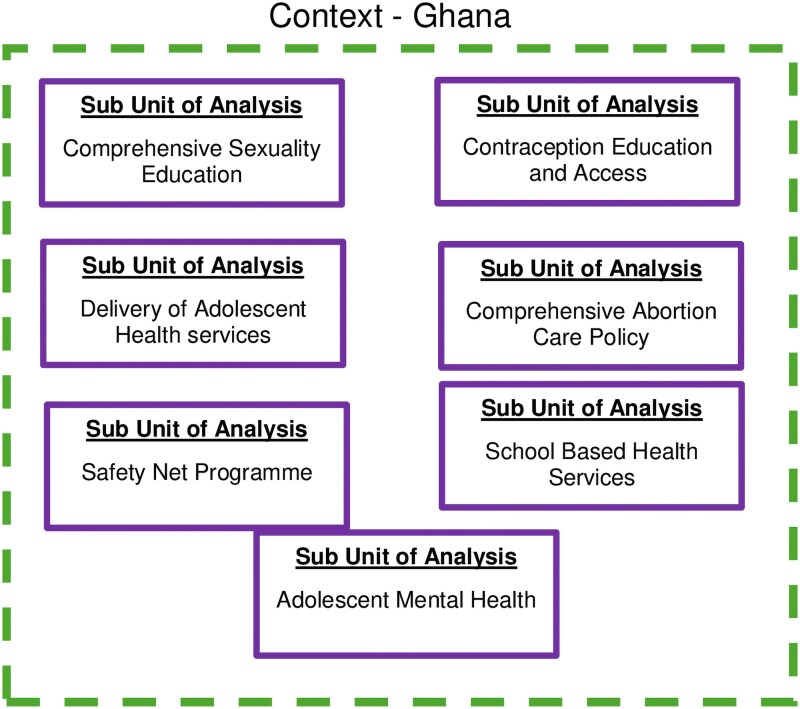
Study design.

We present seven subunits of analysis on a contemporary issue in public policy formulation and/or implementation in AMSRH in Ghana. Our seven cases (subunits of analysis) are the comprehensive sexuality education (CSE), contraceptive education and access, comprehensive abortion care (CAC) policy, delivery of adolescent health services, Safety Net programme, school-based health services policy, and MH. The seven subunits of analysis are summarized in Fig. 2.

In all seven cases, a wide range of actors have been involved from global level stakeholders, such as providers of DAH country offices and representatives, and national level decision-makers and programme managers, to district and community level actors, such as frontline workers, community members, adolescents themselves, and their parents and guardians. Multiple governmental and public sector actors are involved in AMSRH in Ghana, with the health sector playing the central role. Organized along an agency model, the Ministry of Health leads policy development, monitoring, and evaluation coordination across various implementation agencies. The Ghana Health Service (GHS), the primary public healthcare service delivery body and implementation agency, plays a lead role in providing technical advice and leadership in AMSRH policy formulation and implementation. Depending on the issues at stake, several other agencies in health, such as the Mental Health Authority (MHA) and the National Health Insurance Authority (NHIA), a purchaser agency, among others, become key stakeholders. Beyond the health sector, other governmental institutions such as the Ministry of Education and the GES; the Ministry of Gender, Children and Social Protection; the National Population Council (NPC); the National Youth Authority (NYA); and Ghana Aids Commission also play key roles in AMSRH policy agenda setting, formulation, and implementation. DAH agencies included United Nations Population Fund (UNFPA), United Nations Children's Fund (UNICEF), Foreign, Commonwealth & Development Office (FCDO), United States Agency for International Development (USAID), Global Affairs Canada, Japan International Cooperation Agency (JICA), World Health Organization, and the World Bank. International organizations with interests in SRH included Marie Stopes International and the Planned Parenthood of Ghana (PPAG). Other local non-governmental organizations (NGOs) include Alliance for Reproductive Health Rights (ARHR), Hope for Future Generations, Curious Minds, and Basic Needs. Local and international research organizations with an interest in AMSRH included the Guttmacher Institute, Population Council and the Regional Institute for Population Studies (RIPS), University of Ghana.

Actor interest in issues, the use of power, and visibility varied depending on the specific issue, as will be evident in the discussion of the seven cases. Consistently, however, the voice and perspectives of adolescents themselves have been absent from public dialogue, despite the issues directly concerning them. This contrasts with our findings during engagement with adolescents, which revealed their awareness of the issues and their ability to clearly articulate their lived experience, ideas, and opinions.

We have structured the Results section into three clusters of cases characterized largely by (i) major unresolved conflict in the policy formulation and implementation process in the public or bureaucratic arena or both, (ii) consensus across stakeholders, and (3) marginalized agenda (AMH) with many stakeholders not mobilized.

### Cluster 1: Major unresolved contestation in policy formulation and implementation

#### Case 1: CSE

In 2019, plans to introduce a revised CSE programme into Ghana’s school curriculum were widely publicized in print, electronic, and social media (Akorsu 2019, Graphic Online 2019). Rising teenage pregnancy rates and perceived gaps in the existing CSE curriculum prompted the NPA, in collaboration with the GES and supported by UNFPA and other DAH partners to provide age-appropriate and gender-sensitive SRH education to adolescents. The programme was described as ‘a systematic approach to equip young people with the knowledge, skills, attitudes, values they need to determine and enjoy their sexuality-physically and emotionally, individually and in relationships’ (GES, 2019).

However, the announcement triggered widespread suspicion and mistrust. Many viewed it as imposing foreign values that conflicted with Ghana’s conservative norms (Saaka 2024, Chubb *et al.* 2025). Even though the policy document did not refer to LGBTQIA+ issues, the presence of the wording of ‘respect for human rights and diversity with sexuality education affirmed as a right’ as one of the tenets of a CSE programme in the UNFPA Bogota Declaration of 2010 in the document fuelled fears that the programme was an attempt to impose Western values not aligned with majority societal and cultural values and priorities (Saaka 2024, Chubb *et al.* 2025). UNFPA’s funding further deepened mistrust of both the programme and its external backers.

The Ghana Pentecostal and Charismatic Council (GPCC) labelled the programme as ‘Comprehensive Satanic Education’. In a press statement, typical of the widespread societal reaction, the President of the GPCC said: “…we shall not allow any external agency or international government entity to ‘smuggle’ the CSE in the mainstream school curriculum or any other projects or programmes often with the funding of these so-called external interest groups” (GPCC Press Release, October 2019).

Opposition was swiftly mobilized, public, visible, and from powerful societal stakeholders and coalitions, including the political parties in opposition and their members of parliament, religious bodies, parents, and teachers’ unions. Media coverage amplified public debates, framing the CSE programme as undermining traditional values (Akorsu 2019, Appiah 2019 ). Constituents pressured members of parliament whom they felt were supporting the reform, threatening not to vote for them, prompting initial supporters in the governing party to withdraw backing (Dablu 2019 ). The strength of the opposition of the Christian Council, community, and traditional leaders, and the public was profound and led to a halt in the policy formulation and implementation processes (Graphic Online 2019).

Some of the stakeholders in support of the policy indicated that, following the strong public contestation, policymakers have sought to redesign the curriculum to better align with cultural values.

‘We’re closely working with GES, and the outcry over the comprehensive sexuality program was exaggerated. But once it started, we began deliberating on what needs to change to ensure cultural appropriateness’ (KII with National Stakeholder/GHS).

#### Case 2: contraceptive education and access

The GHS and GES are major public sector stakeholders in ASRH service delivery in Ghana. Still, they hold divergent official positions on content and approach for the prevention of unwanted adolescent pregnancy. For the GHS, it was part of desired outcomes for adolescent health service and delivery standards; the GHS seeks to

‘… build capacity of health providers to provide adolescent and youth friendly services and increase contraception use by sexually active adolescents’ [Ghana Reproductive, Maternal, Newborn, Child and Adolescent Health and Nutrition (RMNCAHN) Strategic Plan (2020–2025)].

The GHS thus promotes education on the provision and use of contraception for preventing unwanted adolescent pregnancy. The GES, on the other hand, promotes abstinence-only for adolescents and prohibits the provision of family planning and contraception in schools.

‘In GES, we preach abstinence. We don’t tell the children to engage in sex. We motivate them to abstain while in school. So, abstinence is our keyword. No use of contraceptives while you’re in school. Just live a chaste life!’ (KII with National Stakeholder/GES).

Some GHS frontline providers shared the GES stance and were personally conflicted by their agency’s policy. Unable to openly oppose it, they avoided discussing contraception with adolescents, promoting abstinence instead. The observed effect of this continuing bureaucratic contestation in implementation between the GES and GHS has had varying consequences regarding contraceptive access for adolescents in school and out of school. The contestation was not only bureaucratic. Adolescents themselves expressed divided views in the FGDs. While some endorsed abstinence, others acknowledged sexual activity and advocated for condom distribution.

‘I’m against sharing condoms in school because it tells them to go and do it. It encourages them’ (FGD with in-school adolescent boys).

‘In this 21st century, if you have a boyfriend in SHS [senior high school], and you want a happy relationship with the boy, sex is involved’ (FGD with in-school adolescent girls).

‘My suggestion is that the school should share condoms for the boys so that if you want to have sex, you can prevent pregnancy’ (FGD with in-school adolescent boys).

#### Case 3: CAC policy

In 2020, the GHS, with funding from the Buffett Foundation, institutionalized the CAC within the framework of the Ghana Abortion Law. The RMNCAHN Strategic Plan 2020–2025 states that

‘The Ghana Health Service provides safe abortion and post-abortion care services at its facilities within the context of the Ghana Abortion Law and the policy document that provides guidelines on the provision of these services’ (Ghana RMNCAHN Strategic Plan 2020–2025).

Before this institutionalization, CAC was provided in an *ad hoc* and sporadic manner with intermittent training from NGOs such as PPAG. Institutionalization brought dedicated offices and trained GHS providers.

‘…we didn’t have focused offices with trained persons, and all that. We were doing it with the NGOs who will come to our facilities, and train and help us but now we have GHS service providers who have been trained and who are supposed to render the service…’ (KII with National Stakeholder/GES)

While some providers readily offered CAC, others considered induced abortion incompatible with their personal values and refused to provide the service. The GHS appeared to lack clear processes for reconciling such conflicts. There also appeared to be some stigmatization of those who opted to provide this service.

“A person can conveniently deny a young person a service and there'll be no repercussion. So, they can use their religious stance as a reason for denying services; and it's normal for them. You go into facilities, and they stigmatise providers, who provide comprehensive abortion care…their own colleagues. They have tags for them: ‘this is the abortionist’ …, while per policy, this is a service that they should all be implementing” (KII with Stakeholder/NGO).

This apparent divergence between organizational policy and individual provider values illustrates how policy adoption does not guarantee uniform implementation, with personal positions influencing service delivery outcomes.

#### Case 4: delivery of adolescent health services

In 2020, policy direction for the provision of adolescent health services was subsumed into the RMNCAHN Strategic Plan 2020–2025. The GHS indicated a move towards the integration of all adolescent health services into the primary health care system with the aim of achieving better health outcomes. Youth-friendly corners and separated services for adolescents were going to be phased out.

‘Now the move is to integrate adolescent health services, so it’s no longer targeted at just a few providers but is incorporated into other training programmes to improve attitudes towards adolescents’ (KII with National Stakeholder/GHS).

Stakeholders’ positions on this new policy shift were and remained divided. Supporters argued that youth-friendly corners had become stigmatized as sites for SRH services such as CAC, deterring adolescents. Others within GHS preferred a mixed approach, retaining some adolescent-specific spaces.

‘You need some integration, but there are times when adolescent-specific interventions are necessary because of the unique needs of adolescents’ (KII with National Stakeholder/GHS).

Most NGOs opposed integration, citing underfunding and the risk of further deprioritising adolescent health.

‘I completely disagree. I think pulling everything together into one can be good, but it’s a problem… Already, adolescent health is underfunded in the ministry’s financing mechanism. Now to incorporate it under so many other priority areas of concern under the Health Service… That, for me, is a problem’ (KII with Stakeholder/NGO).

Adolescents themselves articulated shyness when seeking SRH services alongside adults. A potential barrier to accessing SRH services integration across all age groups.

‘Respondent 1: You can go there to meet someone’s mother and tell her that you need a condom. It would be some way …’ (Excerpt of FGD with out-of-school adolescent boys).

At the time of the study, in some districts, while NGOs pushed for the refurbishment of adolescent health corners, state-led actors from the GHS pushed for the integration of adolescent primary care into the general primary care services.

### Cluster 2: consensus in policy formulation and implementation

#### Case 5: Safety Net programme

The Safety Net programme was first piloted by the GHS in collaboration with the Girls Education Unit of the GES and the Department of Social Welfare in 2017. Funding and technical support came from the UNICEF, UNFPA, and Global Affairs Canada. The programme supports adolescent mothers to continue their education through pregnancy and after birth till the first birthday of the child.

‘…Basically, when you are pregnant, we enrol you on the Safety Net. That is if you opt to keep the pregnancy. We follow you up through home visits and help you to acquire some basic things when we get the funding for it; then when you deliver and want to go back to school, we send your name to the girls’ education office and then they follow up and ensure that you go back to school and if you need any social support, we refer you to the social welfare’ (KII with Stakeholder/GHS).

The programme was piloted in 70 districts and scaled up nationally after the pilot ended. Data from our KII interviews with stakeholders indicate general support of the concept and its implementation.

‘Yes so in terms of the targets that we set out, I think we’ve made remarkable progress and with the project ending in 2022, we have even increased our target beyond what was set. There was a baseline and a midline evaluation which shows that yes, there has been progress’ (KII with Stakeholder/DAH).

The collaborative efforts and consensus between stakeholders resulted in effective and timely policy implementation with effective partnerships between the GHS, GES, the Department of Social Welfare, UNICEF, and UNFPA.

‘… a lot of health workers have been trained. We’ve seen over the period, an increase in enrolment of adolescents under the Safety Net programme’ (KII with Stakeholder/DAH).

The government stakeholder also emphasized the need to effectively implement interventions, as that drives funder confidence, drawing attention to the importance of DAH in programme implementation.

‘It is when the one who gave you funds sees that it's working, that's when they scale up. So, you start with 2 districts, the next year you scale up, you get additional support because of the results you're able to produce… For instance, for Safety Net we started with 4 districts in 2017, but now we are working in 70 districts’ (KII with Stakeholder/GHS).

At the end of the piloting phase in 2022, the programme was adopted by the government and scaled up nationally. Implementation was however, less than ideal.

‘We have the staff but the department is not able to play its role effectively because we do not have the [financial]means. …’ (KII with subnational Stakeholder/Social Welfare).

This points to the challenges of sustainably continuing programmes once DAH ends.

#### Case 6: policy and guidelines for school-based health services

In 2018, a Memorandum of Understanding was signed between the GES and GHS, ensuring that the GHS oversaw and managed school sick bays in all senior high schools across the country. The services were undergirded by the Policy and Guidelines for School-Based Health Services with the purpose:

‘…to make health services adolescent and youth friendly. This means that health services at every level should be available, accessible, acceptable, equitable, and affordable for every young client no matter their social or economic status’ Policy and Guidelines for School-Based Health Services (2022 Revised)].

School sick bays were thus manned by nurses from the GHS and offered ASRH services such as menstrual hygiene management, reproductive health assessment, and psychosocial support for pregnant adolescents and adolescent mothers who remained in school. They also provided referral services that linked school clinics with health care facilities.

‘… The school nurse is very nice. She will greet you in a way that makes you feel like going there every day just to go and talk to her… and no matter how ill you are, she will find a way to curb it, but if it is out of her power, you’ll be transferred to the polyclinic’ (FGD with In-School Adolescent Boys).

Even though the GES ensured that all students were registered with the National Health Insurance Scheme (NHIS) to ensure effective implementation as stipulated in the MOU, in practice, the NHIS did not work, and students had to sometimes pay for medication out of pocket or forfeit the service if they did not have money.

‘…I was suffering from malaria. They were pushing me to go to the sickbay but because I did not have money, I said I would not go. So I did not go till now’ (FGD with In-School Adolescent Girls).

All stakeholders saw it as an important and noncontroversial intervention. Implementation challenges were related to resource inadequacy rather than contestation.

### Cluster 3: failure to move beyond agenda setting

#### Case 7: AMH

AMH appeared to suffer from the fact that the issues did not have interest and funding from powerful actors. Providers of DAH, as well as the government prioritized ASRH, which had a well-established and long-standing research and advocacy base as opposed to AMH.

‘…UNFPA’s core business is sexual and reproductive health. So, their organizational makeup gives you an idea of the support they’ll provide’ (KII with National Stakeholder/GES).

Despite featuring as an issue in the policy documents reviewed, AMH was marginalized, and policy formulation and implementation of programmes and interventions to support AMH were not occurring.

‘Very few sponsors find this area interesting. If they don’t support it, researchers won’t pursue it. As a nation, we haven’t reached the point where people research out of personal interest—they go where the funding is’ (KII with National Stakeholder/MHA).

This was despite available research and other evidence of the importance of AMH. For example, the top five causes of years lost to disability in 10–14 year olds include unipolar depressive and anxiety disorders. The picture is similar for 15–19 year olds, with the difference that alcohol use disorders make the list for males (WHO 2024). Government, NGOs, and providers of DAH supporting adolescent health prioritized ASRH as an unquestioned priority in terms of research, policy formulation, implementation, and funding. Adolescents themselves, in our engagement with them, repeatedly brought up MH issues and linked some of them with their SRH issues and wanted interventions to address them.

## Discussion

Understanding how stakeholder power, positions, and interests shape policy processes is critical to explaining policy formulation and implementation in both high-income and LMIC contexts (Gilson and Raphaely 2008, Shearer *et al.* 2016, Campos and Reich 2019, Haelg et al. 2020). Drawing on our study on AMSRH in Ghana, we seek to inform health policy in LMICs, in general, and adolescent health policies analysis, in particular, by addressing the overarching question ‘How does stakeholder power, interest and position influence policy formulation and implementation (or nonimplementation) in LMIC contexts?’. We specifically ask, ‘Who are the actors involved in AMSRH policy formulation and implementation in Ghana?’, ‘What is their power, their interests, and positions in relation to contemporary issues in AMSRH?’, and ‘How do these interact and influence policy formulation and implementation processes?’

Our findings regarding the question ‘who are the actors involved in AMSRH formulation and implementation in Ghana’ reveal a complex policy terrain involving multiple actors from sectors and agencies such as the GHS, GES, bilateral and multilateral providers of DAH active at country level, NGOs, and civil society and communities, with differing levels of power and interests. These differences sometimes catalysed conflict and, at other times, fostered synergies, underscoring the need for early and inclusive stakeholder mapping and analysis. Despite being the primary intended beneficiaries of policy processes, the voices and perspectives of adolescents appeared to be marginalized and often invisible in public implementation. Yet, our interviews and FGDs showed that adolescents possess rich lived experiences, an awareness of the issues, and the ability and desire to articulate their perspectives. The challenge lies in the lack of culturally appropriate opportunities for them to do so. Interventions that empower and support adolescents to share their lived experiences in policy debates, decision-making, and implementation must be done in context-appropriate and sensitive ways that recognize the cultural context and value of respect for the experience and wisdom of older people. Such approaches must navigate the balance between respecting elders’ wisdom and creating space for younger voices. Indeed, this is reflected in the popular Akan proverb ‘*su hye fa yemua*’ meaning ‘do not put the closing centre of a roof in place when only one of the two sides has been completed’. This underscores the importance of listening to all sides before a final decision is made. It is often quoted in trying to resolve and make a fair judgement and decision where there are varying perspectives and differences of opinion.

Adolescent voices are among the many key stakeholder perspectives that need to be clearly heard and understood in policy formulation and implementation decision-making for them. Adolescents, the central beneficiaries of AMSRH policies, must be empowered as partners. Policy and practice must urgently shift to actively involve adolescents in all stages of the policy process to ensure relevant and responsive interventions that align with their lived realities. This can be implemented using youth advisory boards, peer-led outreaches, participatory theatre, and research platforms among others. This observation and our recommendations are of relevance beyond the study context of Ghana. There is global evidence on the marginalization of the lived experiences of adolescents in the health policy-making process that directly affect them, and the need to find how to enable adolescent voice effectively has been observed in studies from other parts of the world (Lopez and Moreira 2013, Jordan *et al.* 2019, Jacobs and George 2022 , Cubas *et al.* 2024 ). Several additional lessons emerge from our findings related to our second subquestion of actor power, interests, and positions in relation to contemporary issues in AMSRH, and how they interact and influence policy formulation and implementation processes that are of relevance in the study setting and beyond. The backlash against the CSE curriculum emphasizes that seemingly dormant stakeholders (in this particular example, religious groups and parents) can prove powerful when mobilized and in their turn mobilize other powerful groups (in this particular case media and parliamentarians). Despite reformers’ strong rationale for CSE, that is, reaching adolescents across school phases and improving informed decision-making, insufficient engagement with key societal actors led to misunderstandings, conflict, and a severe backlash. Effective policy design requires a clear understanding of power dynamics, values, ideas, and perspectives; identifying both active and dormant stakeholders; and recognizing and navigating actual or potential conflicts early.

In the CSE case study, deeply rooted cultural and societal values not adequately taken into account blocked policy implementation despite good intentions from the perspective of the technical stakeholders who crafted and championed the policy. Many issues in adolescent SRH are value-laden and inadequate stakeholder engagement, contextual understanding, and navigation are needed. Our case studies of contraceptive access and CAC show similar issues of contestation albeit not with the high-level public visibility of conflict and mobilization of resistance as in the case of CSE. In both cases, policy implementation faced a more subtle form of institutional resistance, where some frontline providers were conflicted between personal core values and official policy. The resulting modification of implementation arrangements by frontline providers who felt there was a conflict between what they were required to implement in the guidelines (access to contraceptives and CAC) and their personal ideas, opinions, and values around the issue (adolescents must abstain from sexual activity, contraceptives will encourage sexual activity, and the unborn child has a right to life) illustrates the real-world complexities of policy execution. In policy formulation and implementation, especially on value-laden issues, it is important, difficult as it is, to explore how to identify nuances among stakeholders and mechanisms that openly surface and address conflicts in collaborative problem-solving approaches. In addition, framing interventions in terms of shared values and co-developing messaging with faith-based organizations is essential, especially in religious contexts where technically sound policies risk derailment because of unresolved core versus instrumental value conflicts, understandings, and perceptions.

Given value-laden discretion among frontline workers, could policymakers perhaps consider tailoring implementation arrangements? For example, since not all frontline providers were personally conflicted, might it have been possible to assign nonconflicted frontline providers to the provision of services?

In contrast to these cases with unresolved conflict, the implementation of the Safety Net Programme illustrates relatively unhampered implementation within available resources when powerful actors share aligned positions and consensus and collaboration, and the power of actor coalition building and alignment. The role of providers of DAH in supporting AMSRH programmes and policies was, in some cases, positive and, in others, complicated the policy processes we studied. The influence of DAH is profound, shaping policy priorities through financial and technical power. DAH support was positive in enabling the implementation of programmes despite the low-resource context and insufficient resource availability in our cases, with high levels of national and subnational stakeholder consensus and support such as with the Safety Net programme. However, as demonstrated by the CSE case, policies risk rejection when perceived as externally driven and misaligned with local cultural and societal norms. The suspicion of an attempt to impose norms because of financial power helped fuel resistance. Like other actors and stakeholders, providers of DAH in low-resource settings need to understand and navigate context, norms, values, interests, and ideas. Otherwise, DAH, no matter how well intentioned, may fail because of misalignment with dominant local stakeholder ideas, priorities, values, and norms.

Despite adolescent demand for MH services and the evidence of their importance, minimal advocacy and resource allocation left MH marginalized, a negative ‘noncase’ that nonetheless reveals institutional neglect in the policy landscape. In qualitative research, such negative cases are analytically valuable, offering insight into processes of omission and marginalization. Practitioners must frame AMH as an urgent public health priority, mobilizing evidence, champions, and resources to move it from mere agenda setting into formulation and implementation.

Conflict itself is not inherently negative. Potentially, it can trigger awareness, creativity, and better solutions. While it stalled the CSE rollout, it also triggered national dialogue on ASRH and how to address the issues appropriately in context. Where value conflicts arise, structured dialogue processes can bring up underlying concerns. Conversely, the absence of conflict around AMH reflected neglect, not consensus.

Concerning theoretical implications, our study coheres with Lipsky’s street-level bureaucracy theory, evident in the manner in which frontline providers exercised discretion in the implementation of policies (Lipsky, 2010). However, our findings extend this theory by highlighting the moral dimension of such coping mechanisms, suggesting the need for theoretical refinement to account for value-laden actions/positions in policy formulation and implementation.

The conflict over CSE also extends Grindle and Thomas’s (1989) binary view of conflict occurring either in public or bureaucratic arenas. Our data show simultaneous public and bureaucratic contestation: Parliamentarians, initially supportive of the CSE policy in the bureaucratic arena, reversed positions under electoral pressure in the public arena. The overlap of the arenas in this study suggests that conflict can be hybrid and multilayered and calls for theories of policy conflict to evolve to conceptualize overlapping arenas and feedback loops between public sentiment and institutional behaviour. The summary of the subunits of analysis and their arenas of conflict is shown in Table 3.

**Table 3.** Eight subunits of analysis and arenas of conflict.

	Policy agenda issue summary	Stated aim	Arena of conflict and source
1	Comprehensive sexuality education	Provision of age-appropriate and gender-sensitive sexual and reproductive health education to adolescents	Public. Conflict over the cultural appropriateness of the content. Bureaucratic Conflict among key stakeholders on changes to content.
2	Contraceptive education and access	Provision of sexual and reproductive health education and information to adolescents	Bureaucratic. Conflict over content and approach.
3	Comprehensive abortion care	Anyone who meets the criteria and desires it should be provided with abortion care	Bureaucratic. Conflict over induced abortions
4	Integration/mainstreaming of adolescent health services	Integration of all adolescent health services into the general primary health care system	Bureaucratic. Conflict over wanting to have youth-friendly corners for adolescents to provide some if not all services
5	Safety net program	Support to adolescent mothers to continue their education through pregnancy and after birth till the first birthday of the child	Consensus across stakeholders
6	School-based health services	Provision of school infirmaries or sick bays in all the senior high schools across the country.	Consensus across stakeholders
7	You only live once TV drama series	SRH TV drama for adolescents	Consensus across stakeholders
8	Adolescent mental health		

The traditional stages heuristic—problem identification, agenda setting, policy formulation, policy implementation, and policy evaluation—is helpful but limited. Our seven cases demonstrate blurred boundaries and iterative processes, supporting Sabatier’s (1991) critique of linear models. For example, while MH remains at the agenda-setting stage, programmes like the Safety Net are in advanced implementation. Policy processes are best understood as a continuum rather than as discrete phases. These realities reinforce the need to conceptualize policy processes as dynamic, nonlinear, and deeply embedded within broader socio-political contexts.

## Conclusion

This study emphasizes that AMSRH policy processes in Ghana and, by extension, similar LMIC settings are shaped by complex, interrelated dynamics of actor interests, power, structural influence, and societal values, which are neither static nor confined to discrete stages or arenas. Effective policy formulation and implementation require not only technical soundness but also a deep understanding of these dynamics. Strengthening AMSRH policies will demand inclusive value-sensitive engagement, and iterative feedback with all relevant actors, with an emphasis on amplifying the voice of adolescents alongside strategies to navigate moral contestations, foster stakeholder alignment, and address areas of policy neglect.

## Data Availability

The anonymized interview and focus group discussion transcripts analysed for this study are available upon request and not available in a public repository due to the sensitive nature of the issues discussed.

## Reflexivity statement

The authors include five women and one man with professional experience spanning from Professorship to Master’s level. Four of the authors are from Ghana, with one from Canada based in Ghana and one from Tajikistan based in the UK with substantial experience of research in LMICs. The study benefitted greatly from the vast expertise of the senior author in Ghana who has had long interactions with the health systems actors, which contributed deeper insights but did not constitute a conflict of interest for the reported analysis. The social locations of the authors impacted on the research and care were taken to practice reflexivity, through writing field notes and regular team meetings.
